# Intensive longitudinal study of newly graduated nurses’ quick returns and self-rated stress

**DOI:** 10.5271/sjweh.3962

**Published:** 2021-06-29

**Authors:** Anna Dahlgren, Philip Tucker, Aleksandra Bujacz, Elin Frögéli, Ann Rudman, Petter Gustavsson

**Affiliations:** Division of Psychology, Department of Clinical Neuroscience, Karolinska Institute, Sweden; Stress Research Institute, Stockholm University, Stockholm, Sweden; Psychology Department, Swansea University, Swansea, United Kingdom

**Keywords:** diary study, recovery, rotating shift, shift work

## Abstract

**Objective:**

Little is known about the relationship between quick returns (QR) – shift combinations that result in inter-shift rest periods <11 hours) and stress. The current study examined whether variations in the frequency of QR, both between and within individuals, were associated with changes in self-rated stress.

**Methods:**

A questionnaire was sent weekly to newly graduated nurses during the first 12 weeks of work. Stress was measured with four items from the Stress-Energy Questionnaire on a scale from 1 “not at all” to 5 “very much” [mean 2.65, standard deviation (SD) 1.08]. Shifts worked in the past week were reported and QR were identified by evening-morning shift combinations (mean 0.98, SD 0.90 per week). In total, 350 persons were included in the analysis (3556 observations). Data were analyzed with a multilevel residual dynamic structural equation model (RDSEM) using Bayesian estimation procedures.

**Results:**

There was no between-person effect of QR on stress averaged across measurement occasions (0.181, 95% CI -0.060–0.415). However, there was a small within-person effect of QR (0.031, 95% CI 0.001–0.062), meaning that more QR during a given week, compared to that person’s average, was associated with an increase in their level of stress during that week.

**Conclusions:**

Nurses were likely to report increased stress during weeks in which they worked more QR. Intervention studies are needed to determine whether the relationship is causal.

Quick returns (QR) – shift combinations resulting in rest periods <11 hours – are widespread throughout Swedish healthcare with nurses commonly finishing afternoon shifts at 21:30 hours followed by a morning shift at 06:45 hours, allowing just over 9 hours to manage travel, food intake, hygiene, social obligations and sleep. Anecdotally, QR contribute to continuity of care since nurses are already acquainted with their patients when they return to start their morning shift. This, in turn, may reduce workload as less time is needed to read patient records. QR also allow nurses to compress their work weeks, allowing longer times off. However, QR also seem to be associated with insufficient recovery due to short sleeps, impaired sleep quality, insomnia, longer sleep latencies and greater levels of fatigue, which in turn may increase risk for sickness absence and injuries (1–3).

Lack of recovery contributes to sustained activation of the stress system, ie, allostatic load (4). Thus, QR are a potential cause of stress due to the limited opportunities they afford for recovery. However, there is limited evidence of a relationship between QR and stress. A study of midwives found that an intervention involving a reduction of QR led to decreased reports of mental strain and stress, although the results were hard to interpret as multiple aspects of the scheduling arrangements were changed simultaneously (5). More recently, a cross sectional study of doctors found a positive association between frequency of QR and reported stress (6). However, in contrast to these findings, a diary study of nurses found no within-person differences in stress levels between morning shifts that were preceded by either a QR or no QR (7).

Newly graduated nurses experience high levels of stress due, in part, to being new to the profession. As many as 20% of newly graduated nurses experience high levels of burnout symptoms during their first five years in the profession (8). Thus, early career nurses may be especially vulnerable to occupational factors that impair the ability to deal with stress, including QR. We have shown that newly graduated nurses can have difficulties unwinding after work during a QR, especially during periods of high workload (9). Rumination was thought to play a key role, contributing to sustained activation of the nurses’ stress systems and impaired recovery during QR.

The aim of the present study was to use an intensive longitudinal design to determine whether variation in QR, both within and between individuals, was associated with self-rated stress in newly graduated nurses.

## Method

An intensive longitudinal study design with weekly data collections over 12 consecutive weeks was used. Participants were recruited in four rounds between 2015–2018. Eligible participants were nursing students who were about to graduate and commence their first position as registered nurses. In 2015 and 2016, information was emailed via all universities holding nursing programmes to all nursing students and presented on digital study platforms. In 2018, information was emailed and presented verbally by the researchers at 12 universities. In addition, on all occasions, an advertisement was posted on the research group’s Facebook page. We do not know how many received the information and fulfilled the inclusion criteria. However, 4099 students were enrolled at the final semester at the time of data collection. In total 409 students chose to participate, 365 completed the baseline questionnaire and 350 participated with longitudinal data during workdays. All participants gave informed consent to join the study, which had ethical approval (2014/1531-31/5, 2017/543-31/5).

A digital survey-tool (Artologik) was used for collecting data weekly during 12 consecutive weeks, starting in the first or second week of employment. Surveys were sent via email to participants on the same day and time of the week. Approximately 83–95% (across the different rounds of recruitment) answered before reminders were sent to participants who had not responded to the survey within four days. Each survey was active for one week until the next survey was sent out. On average the participants answered ten surveys (SD 1.63).

Stress was measured with three items from the Stress-Energy Questionnaire (10). Participants answered the questions “During the past week when you have been working, how often have you felt… tense?, stressed?, and pressured?”. The answers were given on a scale ranging from never=1 to always=5. The scale had high internal consistency, alpha=0.87, mean 2.65, SD 1.08. QR were identified from shift combinations with evening shifts followed by a morning shift. Range was 0–4 QR shifts per week (mean 0.98, SD 0.90).

Data were analyzed with a multilevel residual dynamic structural equation model (RDSEM) using Bayesian estimation procedures in Mplus version 8.4 (11). The method integrates time-series analysis (modeling the lagged relations in repeated measures), multilevel modelling (allowing for simultaneous modelling of individual processes and differences between individuals), and structural equation modelling (allowing for latent variables modelling). Moreover, the use of Bayesian estimation provides credibility intervals based on a posterior distribution that are more intuitive to interpret (11). The effects of QR on stress were modelled on both between-person (averaged across measurement occasions) and within-person (between measurement occasions) levels. Both predictor and outcome variables were partitioned into within-between components using latent mean centering (12). Time-invariant covariates (gender, age, and cohort) were included to control for between-person variability in stress intercepts. Additionally, a linear development trend over time was modelled, accounting for autoregressive correlation of residuals.

## Results

The analytical sample consisted of 350 participants (mean age 28.52, SD 6.67 years; 88.3% women) and 3556 measurement occasions. Table 1 highlights the effects estimated in the model relevant to the aim of the study. Stress levels differed between persons when averaged across measurement occasions, such that participants in the more recent cohorts reported higher stress. The general trend over time was negative, meaning that stress decreased over the first 12 weeks of employment. No between-persons effect of QR on stress levels averaged across measurement occasions was detected (0.181, 95% CI -0.060–0.415). This means that those nurses who worked more QR on average over the 12 weeks period did not report significantly higher stress levels. The within-persons effect of QR on stress at a given measurement occasion was (0.031, 95% CI 0.001–0.062), indicating that more QR during a given week, compared to a person’s average, increased the stress level during that week. Model based variances of QR for the between- and within-persons were 0.212 and 0.571, respectively; residual model based variances of stress were 0.356 and 0.474, respectively. The within-level R-square was 0.282 (95% CI 0.245–0.316), which suggests that about one third of the within-person variance of stress was explained by within-person QR variability (on average across subjects), together with a time trend.

**Table 1:** Unstandardized estimates and 95% confidence intervals (CI) for the chosen effects in the multilevel RDSEM model. [QR=quick returns]

Stress on	Posterior median	95% CI
	
Gender	-0.022	-0.311–0.271
Age	-0.015	-0.030–0.000
Cohort	0.135	0.012–0.257
Time	-0.017	-0.028– -0.005
QR between	0.181	-0.060–0.415
QR within	0.031	0.001–0.062

## Discussion

Nurses reported increased stress during weeks in which they worked more QR. One possibility is that the relationship between QR and stress is mediated by impaired recovery. Quick returns limit the opportunity for recovery between work shifts. Insufficient recovery between work shifts results in the worker returning to work the next day in a sub-optimal state, such that they need to invest additional compensatory effort in order to perform adequately at work (13). The additional compensatory effort that accompanies QR may provoke a stress response. Epstein et al (9) reported that nurses experienced little fatigue during shifts following QR but intense fatigue after those shifts, indicating that stress activation may be masking the underlying fatigue at work. At the same time, the stress response may further inhibit recovery. A sustained stress activation and an inability to unwind after work (eg, due to rumination about the day’s experiences and anticipation of the day to come) can lead to longer sleep latency and more fragmented, poorer quality sleep (9, 13). A vicious circle then ensues whereby stress increases the need for recovery, while at the same time high stress leads to impaired recovery, also referred to as the recovery paradox. Cutting down on recovery behaviors such as social life, hobbies, physical exercise etc. has been reported among newly graduated nurses (9, 14), which might fortify such a vicious circle.

An alternative explanation of the association between QR and stress is that nurses may be more likely to work QR during periods with high demands, for example, as a result of being required to work additional shifts. Thus, high demands on the service may be an underlying cause of both increased frequency of QR and increased stress among nurses. This scenario could account for the observed pattern of results, without the need to invoke a causal relationship between QR and stress. However, in additional analyses (not shown), we found only weak correlations between self-rated workload and frequency of QR (r<0.10 for within and between subjects). Thus, an arguably more likely scenario is that QR do increase stress, and that this effect is intensified during periods of high demand on the healthcare service.

It is also possible that the association between QR and stress is mediated by work–family interference, especially if the QR are associated with schedule changes at short notice. We lacked the information to examine this possibility, but future research in this area should include measures that capture stress stemming from work–home interference, as well as indicators of short notice changes to work schedules.

The observed within-subject effects of QR on stress were significant, with the model accounting for about one third of the observed variance in reported stress. Nevertheless, the effects were relatively small, which may indicate that the impact of a QR on acute stress is relatively short lived. If so, our measure of stress experienced over the course of the entire week may be somewhat insensitive to such acute effects. Future research should employ even more intensive (eg, daily) measurements of stress and work hours, so that effects in the immediate aftermath of a QR can be studied. The lack of between-persons effect was not expected but may have been due to the insensitivity of the comparison, relative to the within-persons effect. As well as the potential for unmeasured confounds, the sensitivity to the acute effects of QR may have been diluted by relying on measurement over a 12-week period (see above).

Strengths of current study included the low levels of missing responses to the weekly questionnaires (on average participants answered 10 of 12 questionnaires) and the use of an intensive longitudinal design with a large sample, resulting in a large number of measurement points. Moreover, studying nurses at the beginning of their career reduces the risk of ‘healthy worker’ selection effects, ie, when study samples become biased as a result of vulnerable workers quitting positions that involve demanding shift combinations. The frequency of quick returns was similar to a previous study of nurses (7), suggesting that the current findings are generalizable to other healthcare systems where QR are present (eg, in Scandinavia). However, the findings may be less generalizable to other groups, especially outside healthcare settings, as newly graduated nurses experience a unique work situation and demands. While there is some uncertainty regarding the response rate to the invitation to the study, this is unlikely to have had a significant impact on the veracity of the observed within-person changes over time.

In conclusion, nurses were likely to report increased stress during weeks in which they worked more QR. Intervention studies are needed to determine whether the relationship is causal.
